# A virtual One Health Community of practice in West Nile sub-region, Uganda: Implementation experience and participation trends

**DOI:** 10.1016/j.onehlt.2026.101471

**Published:** 2026-06-09

**Authors:** Dickson Akankwatsa, Maureen Kesande, Ivan Segawa, Gilbert Aniku, Dathan Byonanebye

**Affiliations:** a Infectious Diseases Institute, College of Health Sciences, Makerere University, Kampala, Uganda; b Arua Regional Referral Hospital, Ministry of Health, Uganda; c West Nile Regional Emergency Operations Centre, Uganda

**Keywords:** One Health, Community of practice, Multi-hazard risk assessment, Uganda, SWOT analysis, Virtual engagement

## Abstract

Uganda faces recurrent and emerging health threats due to its proximity to the biodiverse Congo Basin. A multi-hazard risk assessment using the WHO Strategic Toolkit for Assessing Risks identified and prioritized nine public health hazards in the West-Nile sub-region, Uganda. Recognizing that most hazards had significant One Health implications, a One Health Community of Practice (OHCoP) was established to enhance cross-sectoral collaboration and coordination. Based on a Strengths, Weaknesses, Opportunities, and Threats analysis, virtual engagement was selected as the most feasible, cost-effective, and scalable dissemination strategy. Between April 2023 and September 2024, 11 of 18 planned virtual sessions were conducted, with a median attendance of 33 (range: 20–145), predominantly human health professionals (94%). Although the virtual OHCoP proved a practical and low-cost model for cross-sectoral knowledge exchange, stronger inclusion of non-human health sectors will require improved digital literacy, professional incentives, blended formats, and institutional commitment to joint planning and decision-making.

## Highlights

• Eleven of eighteen One Health Comminity of Practice sessions completed between 2023 and 2024.
• Human health professionals dominated participation (94%).
• Digital illiteracy and poor connectivity limited cross-sectoral attendance.
• Virtual CoPs offer a cost-effective, scalable model for One Health collaboration.
• Participation trends highlight structural imbalance in One Health engagement across sectors.

## 1 Introduction

The world continues to face emerging and recurrent health threats that transcend disciplinary and geographical boundaries [1]. Like many low- and middle-income countries, Uganda experiences seasonal public health emergencies, such as floods and infectious disease outbreaks, driven by environmental and climatic changes [2]. Addressing these challenges requires a risk-based, multi-sectoral approach that optimizes resources and guides preparedness and response based on evidence and contextual priorities [3].

To strengthen this capacity, Uganda developed a National Multi-Hazard Emergency Preparedness and Response Plan in 2016, coordinated by the Office of the Prime Minister (OPM) [4]. While the plan emphasized an all-hazard approach integrating human, animal, and ecosystem health, its early implementation relied heavily on ad hoc risk profiling and expert opinion [5]. To enhance comparability and evidence use, Uganda adopted the WHO Strategic Tool for Assessing Risks (STAR), providing a structured framework for multi-hazard risk profiling and prioritization [6].

Uganda's vulnerability to multiple hazards is compounded by its proximity to the biodiverse Congo Basin, a hotspot for zoonotic and emerging infections, and by its open refugee policy, hosting over 1.7 million refugees from neighboring countries [7]. Refugee settlements in Uganda face constrained sanitation and health services, increasing risks of outbreaks of cholera, malaria, measles, and typhoid, all of which were identified as high-risk hazards in the 2019 national STAR assessment. Additional hazards, including meningitis, viral hemorrhagic fevers, drought, floods, and landslides, further stress local response capacity [3].

To contextualize national findings, the Ministry of Health (MoH), in partnership with the OPM, the West Nile Public Health Emergency Operations Centre (WNPHEOC), and the Infectious Diseases Institute (IDI), conducted a multi-hazard risk assessment for the West Nile Sub-region in March 2023. The assessment identified Meningitis, Viral Hemorrhagic Fevers, Refugee influxes, Anthrax, Rabies, Malaria, Plague, Diarrheal diseases, Cholera, Typhoid, and Measles as key threats, most of which necessitated coordinated One Health action across sectors.

In response, partners established a One Health Community of Practice (OHCoP), a multidisciplinary platform for knowledge exchange, joint learning, and cross-sectoral coordination across One Health domains to be implemented virtually [8], [9]. This paper describes the implementation experience and participation trends of the virtual OHCoP, drawing lessons for strengthening cross-sectoral collaboration in resource-limited settings.

## 2 Methods

**Fig. 1:**
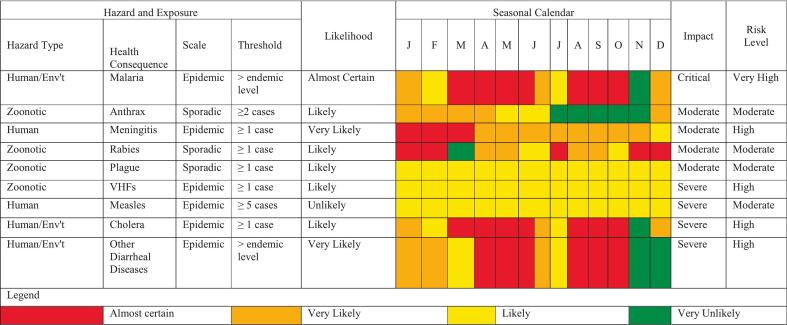
Summary of prioritized hazards for West Nile sub-region March 2023. Seasonal Calendar has been split by month from January to December. Env't: Environment. VHFs: Viral Hemorrhagic Fevers.

A descriptive, implementation study using a participatory approach was conducted in the West Nile sub-region of Uganda from April 2023 to September 2024. This study followed a multi-hazard risk assessment using the WHO STAR (2021) [6], to systematically identify, profile and prioritize public health hazards (Fig. 1).

Following hazard prioritization, a physical stakeholders' meeting was convened, involving the WNPHEOC chair, district health officers (human, animal, and environment), surveillance officers, and the IDI team in the region, to review the findings and discuss appropriate dissemination strategies. Most prioritized hazards exhibited cross-sectoral One Health relevance, indicating that effective management required coordinated actions across human, animal, and environmental health sectors.

Stakeholders discussed three dissemination options: (1) district-level meetings, (2) virtual meetings, and (3) regional-level workshops. The options were evaluated using a SWOT (Strengths, Weaknesses, Opportunities, and Threats) analysis, considering cost, scalability, inclusivity, and coverage (Supplementary Table S1). The virtual engagement model was selected as the most feasible, cost-effective, and scalable approach, considering limited resources, wide geographical coverage, and the need for continuity of the engagements.

The One Health approach [8], [10] informed the formation of a virtual OHCoP, an online platform for cross-sectoral knowledge exchange and coordination in preparedness and response activities. Monthly virtual sessions were held via Zoom, featuring expert-led interactive presentations, discussions, and feedback on priority hazards and operational challenges identified during the STAR assessment. For hazards with seasonal patterns, the monthly topics were aligned with the expected hazard for that period. Sessions were held on the first Thursday of each month, from 14:00 to 16:00 h. The subject matter experts adapted the presentation materials from resources developed by MoH, in collaboration with IDI, to ensure cross-sectoral relevance. Session invitations were disseminated through WhatsApp, institutional mailing lists, and partner networks. Participation was voluntary and open to professionals from human, animal, and environmental health sectors, with each session targeting 200 participants. To facilitate participation, IDI reimbursed each participant with USD 2.8 for internet data costs.

We assessed session attendance (by topic), sectoral representation and geographical reach. Participant engagement was operationalized primarily through attendance, which served as the main indicator of active participation and uptake of the virtual platform across the One Health sectors. This was because attendance provides an objective, consistently recorded and comparable measure of platform utilization in virtual engagement settings. Attendance was recorded for each session using a structured Google Form that captured participants' professions, districts, and session topics. Data were cleaned in Microsoft Excel and analyzed in R (version 4.4.1). Participation statistics were summarized using proportions and medians, while Kruskal-Wallis and Fisher's exact tests were used to compare participation across topics and sectors.

## 3 Results

Between April 2023 and September 2024, 11 of 18 planned OHCoP sessions (61%) were conducted (Table 1), drawing 548 cumulative participants from 13 districts of the West Nile sub-region, recorded across all sessions. Each session targeted 200 participants; however, attendance was low, with a median attendance of 33 (range: 20–145) per session. Attendance fluctuated considerably across topics, with human health professionals forming the majority of participants (94%). Participants during session feedback time attributed this low attendance to digital illiteracy, lack of appropriate devices, and unreliable internet connectivity. Provision of incentives, such as certificates for recognition and professional credits, was mentioned as a way to improve attendance.

**Table 1:** Summary of attendance across the session topics and professional categories.

Month year	Topic	Total attendees, n (% = n/200)	Human health, n (%)	Animal health, n (%)	Environmental health, n (%)
April 2023	Introducing the concept	20 (10.0)	17 (85)	2 (10.0)	1 (5.0)
May 2023	Anthrax	28 (14.0)	26 (92.9)	2 (7.1)	0 (0.0)
June 2023	Malaria	20 (10.0)	19 (95)	1 (5)	0 (0.0)
July 2023	Meningitis	32 (16.0)	32 (100)	0 (0.0)	0 (0.0)
Aug 2023	ESA	22 (11.0)	21 (95.5)	1 (4.5)	0 (0.0)
Sep 2023	Cholera	33 (16.5)	33 (100)	0 (0.0)	0 (0.0)
Oct 2023	Typhoid	42 (21.0)	40 (95.2)	1 (2.4)	1 (2.4)
Jan 2024	Rabies	86 (43.0)	64 (74.4)	21 (24.4)	1 (1.2)
Feb 2024	Anthrax	48 (24.0)	48 (100)	0 (0.0)	0 (0.0)
May 2024	Viral conjunctivitis	145 (72.5)	143 (98.6)	2 (1.4)	0 (0.0)
Sep 2024	Measles	72 (36.0)	71 (98.6)	1 (1.4)	0 (0.0)
Total		**548**	**514 (93.8)**	**31 (5.7)**	**3 (0.5)**

Note. ESA: Enhanced Situation Awareness.

Among all sessions, the rabies topic attracted the highest cross-sectoral engagement (74% [64/86] human, 24% [21/86] animal, 1% [1/86] environmental health professionals), while the one on viral conjunctivitis had the highest overall attendance (145 participants). Other sessions, such as meningitis, cholera, and anthrax, were attended exclusively by human health professionals.

Statistical analysis showed no significant difference in median attendance across session topics (Kruskal-Wallis, *p* = 0.94). However, professional representation varied significantly by topic (Fisher's exact, *p* < 0.001), indicating that session relevance influenced participation across sectors.

## 4 Discussion

This evaluation examined the implementation and participation trends of the virtual OHCoP in Uganda's West Nile sub-region between April 2023 and September 2024. Despite a modest session completion rate (61%), the OHCoP demonstrated the feasibility of a low-cost, scalable mechanism for fostering cross-sectoral dialogue on emerging and remerging health threats. By leveraging digital platforms, the initiative expanded its geographical reach and facilitated knowledge exchange across district and sectoral boundaries. However, participation was highly uneven, with human health professionals dominating the sessions and the animal and environmental health sectors being underrepresented.

The predominance of human health cadre participation (94% overall) reflects a longstanding structural imbalance in One Health implementation, in which human health tends to receive greater attention, funding, and visibility than animal and environmental health [10], [11]. Similar patterns are observed across sub-Saharan Africa, where One Health activities remain anchored in public health institutions rather than shared governance platforms [12], [13] The limited representation of animal and environmental health professionals in the OHCoP may therefore reflect broader institutional arrangements within One Health systems, where opportunities for joint planning, training, and decision-making are often concentrated within the human health structures [12], [13], [14], potentially constraining incorporation of animal and environmental health sectors' perspectives that are essential for comprehensive preparedness and response to complex public health threats. This implies the need for policy and implementation frameworks that support equitable cross-sectoral engagement.

This finding is consistent with broader evidence that operationalization of One Health still leans toward biomedical approaches despite policy-level recognition of its cross-sectoral nature [14]. The repeated occurrence of human-centered outbreaks such as COVID-19, Ebola, and cholera has reinforced the prioritization of the human health topic [10], [14]. Moreover, the virtual format may have inadvertently favored participants already familiar with digital engagement, primarily the clinicians and public health officers accustomed to online continuous professional development, similar to findings by other scholars [15], [16].Overall session attendance was low (median of 33), which was attributed to a lack of digital literacy, limited access to appropriate devices, and unreliable internet connectivity, issues that are consistent with challenges of e-learning in resource-limited settings [15], [16]. Although a modest post-session internet bundle reimbursement was provided, delayed disbursement and poor connectivity in remote areas likely discouraged real-time engagement. Addressing these barriers will require integration of digital literacy into professional training across sectors and exploring blended (virtual and in-person) learning modalities, especially in rural districts [17].

The rabies session attracted the highest cross-sectoral engagement, while topics around viral conjunctivitis and measles recorded the highest overall attendance. Significant differences in attendance of session topic by professional background suggest that the attendance was influenced more by sectoral interest than general One Health enthusiasm. The rabies session called both human and animal health professionals to action, aligning with prior studies that have emphasized it as a model for One Health collaboration [11]. On the contrary, limited engagement in anthrax sessions and other zoonoses may reflect variable awareness and perceived relevance [13].

Participants suggested that incentives such as professional development credits, certificates, or formal recognition could improve and sustain attendance, particularly among underrepresented sectors. This observation is consistent with previous evidence that non-financial incentives can strengthen professional engagement [18]. Sustaining One Health efforts will require institutional and policy-level commitment to joint planning, budgeting, and training across ministries and sectors [13], [14]. Embedding One Health concepts within pre-service training curricula could promote collaboration and shared responsibility from early career stages [10].

The virtual OHCoP minimized logistical constraints, expanded geographical reach, consistent with other studies showing that virtual CoPs efficiently connect professionals across distances and reduce travel burdens [17], [19], [20]. Nonetheless, sustained participation depends on deliberate facilitation, institutional support, and reliable connectivity, emphasizing the importance of balancing technical feasibility with equitable representation to achieve inclusivity.

The West Nile experience demonstrates that virtual CoPs are a feasible, low-cost, and scalable model for strengthening multi-sectoral coordination on health threats in resource-limited settings. Ensuring equitable participation will require improved digital literacy, the provision of professional incentives, and the adoption of blended learning formats to enhance inclusion of animal and environmental health professionals.

Among the limitations, the paper utilized data from a small number of completed sessions, had unequal participation across sectors, and relied on self-reported data. In addition, attendance was captured cumulatively rather than at the individual level, limiting the ability to assess unique participation, continuity of engagement and the extent to which same individuals contributed across sessions. Furthermore, the virtual format may have introduced variability in participation due to differences in internet access, digital literacy and other competing professional demands. Future evaluations should include formal qualitative assessments of learning, collaboration, and impact to better understand how virtual platforms can institutionalize One Health coordination.

## CRediT authorship contribution statement

**Dickson Akankwatsa:** Writing – review & editing, Writing – original draft, Visualization, Validation, Methodology, Formal analysis, Data curation, Conceptualization. **Maureen Kesande:** Writing – review & editing, Visualization, Validation, Project administration, Data curation. **Ivan Segawa:** Writing – review & editing, Visualization, Validation, Data curation. **Gilbert Aniku:** Writing – review & editing, Visualization, Validation, Methodology. **Dathan Byonanebye:** Writing – review & editing, Validation, Project administration, Methodology.

## Informed consent and patient details

Ethical approval for this activity was obtained through the Ugandan Ministry of Health, as part of routine preparedness strengthening and quality improvement efforts, and was considered exempt from formal institutional review board approval.

## Funding sources

Not applicable.

## Declaration of competing interest

The findings and conclusions in this report are those of the author(s) and do not necessarily represent the official position of the funding agencies.

## Data Availability

The datasets used to produce this work are available from the corresponding author on reasonable request.

## Appendix A Supplementary data

Supplementary Table S1 SWOT analysis of the multi-hazard risk assessment data dissemination options.
