# Supplementary material for: A virtual One Health Community of practice in West Nile sub-region, Uganda: Implementation experience and participation trends
Source: One Health. 2026 Jun 9;22:101471. doi: 10.1016/j.onehlt.2026.101471 (PMC13273853; doi:10.1016/j.onehlt.2026.101471)
Supplement: Supplementary Table S1 — SWOT analysis of the multi-hazard risk assessment data dissemination options. [file mmc1.docx]

**Supplementary materials**

Supplementary Table S1: SWOT analysis of dissemination options

| **Option** | **Strengths** | **Weaknesses** | **Opportunities** | **Threats** |
| --- | --- | --- | --- | --- |
| District-level meetings (23,612.4USD/85m UGX) | - Key stakeholders involved first hand - Creates sense of district ownership & contextual understanding of the findings - Allows tailored discussions and actions | - Miss end users of the information - One-off event - Costly, involves venue hire, meals, fuel, facilitation for facilitators, and facilitation for participants (SDA, transport refund) | - Potential to integrate findings into local development plans. - Alignment with national decentralization policies and donor interest. - Full engagement of key leadership | - Real end users are missed. - Staff turnover may affect continuity - Budget constraints from the partners supporting the activities. |
| Virtual engagement (10,000USD/36m UGX). | - Cost effective (participants would only need data bundle refund) & cost spread cross 1.5 years - Time efficient - Enables broad participation across districts at same time - Easy to document proceedings - Participate at own comfort | - Connectivity challenges in rural areas - Reduced interpersonal interaction and engagement - Limited attendance by stakeholders without smart phones or those not used to digital tools | - Growing adoption of digital tools in government and health sector - Potential partnerships with e-learning providers | - Internet instability and power outages - Risk of disengagement of participants - Excludes stakeholders without reliable ICT access. |
| Regional-level workshops (71,913.9 USD/258.9m UGX). | - Encourages cross district learning and sharing of best practices - Strengthens regional collaboration | - May dilute district specific issues and priorities - Requires coordinating across multiple administrative units which may not be easy. - Misses real end users/beneficiaries of the information - Travel costs are way too high (participants would require perdiem, transport refund), and also venue hire. - One-off event | - Regional health initiatives offer platform for collaboration - Opportunity to advocate for MHRA priorities in regional preparedness frameworks | - Risk of uneven representation from some districts - Possible logistical challenges in convening participants - Competing priorities may affect attendance |

MHRA: Multi-hazard Risk Assessment, UGX: Ugandan shillings, USD: US Dollars, SDA: Safari Day Allowance, ICT: Information and Communication Technology
